# Control of HIV-1 replication *in vitro* by vaccine-induced human CD8^+^ T cells through conserved subdominant Pol epitopes

**DOI:** 10.1016/j.vaccine.2015.12.021

**Published:** 2016-02-24

**Authors:** Tina Ahmed, Nicola J. Borthwick, Jill Gilmour, Peter Hayes, Lucy Dorrell, Tomáš Hanke

**Affiliations:** a The Jenner Institute, Nuffield Department of Medicine, University of Oxford, Oxford OX3 7DQ, United Kingdom; b Human Immunology Laboratory, International AIDS Vaccine Initiative, London SW10 9NH, United Kingdom; c Faculty of Medicine, Imperial College, London SW7 2AZ, United Kingdom; d Nuffield Department of Medicine, NDM Research Building, University of Oxford, Oxford OX3 7FZ, United Kingdom; e Oxford NIHR Biomedical Research Centre, University of Oxford, Oxford, United Kingdom; f International Research Center for Medical Sciences, Kumamoto University, 2-2-1 Honjo, Chuo-ku, Japan

**Keywords:** Conserved region vaccine, Virus inhibition assay (VIA), HIV-1 vaccines, Human CD8^+^ T cells, T cell epitopes, T cell vaccines, HIVconsv, HIV-1 derived immunogen based on conserved alternating clade consensus sequences, VIA, virus inhibition assay, HLA, human leukocute antigen, HIV-1, human immunodeficiency virus type 1, SIV, simian immunodeficiency virus, PBMC, peripheral blood mononucleated cells, PHA, phytohaemagglutinin

## Abstract

**Objective:**

The specificity of CD8^+^ T cells is critical for early control of founder/transmitted and reactivated HIV-1. To tackle HIV-1 variability and escape, we designed vaccine immunogen HIVconsv assembled from 14 highly conserved regions of mainly Gag and Pol proteins. When administered to HIV-1-negative human volunteers in trial HIV-CORE 002, HIVconsv vaccines elicited CD8^+^ effector T cells which inhibited replication of up to 8 HIV-1 isolates in autologous CD4^+^ cells. This inhibition correlated with interferon-γ production in response to Gag and Pol peptide pools, but direct evidence of the inhibitory specificity was missing. Here, we aimed to define through recognition of which epitopes these effectors inhibit HIV-1 replication.

**Design:**

CD8^+^ T-cells from the 3 broadest HIV-1 inhibitors out of 23 vaccine recipients were expanded in culture by Gag or Pol peptide restimulation and tested in viral inhibition assay (VIA) using HIV-1 clade B and A isolates.

**Methods:**

Frozen PBMCs were expanded first using peptide pools from Gag or Pol conserved regions and tested on HIV-1-infected cells in VIA or by individual peptides for their effector functions. Single peptide specificities responsible for inhibition of HIV-1 replication were then confirmed by single-peptide expanded effectors tested on HIV-1-infected cells.

**Results:**

We formally demonstrated that the vaccine-elicited inhibitory human CD8^+^ T cells recognized conserved epitopes of both Pol and Gag proteins. We defined 7 minimum epitopes, of which 3 were novel, presumably naturally subdominant. The effectors were oligofunctional producing several cytokines and chemokines and killing peptide-pulsed target cells.

**Conclusions:**

These results implicate the use of functionally conserved regions of Pol in addition to the widely used Gag for T-cell vaccine design. Proportion of volunteers developing these effectors and their frequency in circulating PBMC are separate issues, which can be addressed, if needed, by more efficient vector and regimen delivery of conserved immunogens.

## 1 Introduction

CD8^+^ T cells play an important role in the control of HIV-1 infection [1]. In humans, this is supported indirectly by the kinetics of early partial control of viremia as the first CD8^+^ T-cell responses appear, extensive virus escape in targeted epitopes and by genome association studies showing protective effects of certain HLA class I allotypes [2], [3], [4], [5], [6]. Model infection of rhesus macaques with SIV provided a clear demonstration that T cells can both protect from [7] and clear [8] SIV infection. Thus, induction of highly effective cytotoxic T cells by vaccination has the potential to significantly contribute to a successful prophylaxis by complementing antibody vaccines while these are suboptimal and to immunotherapy by killing cells with reactivated virus.

CD8^+^ T cells recognize and kill HIV-1-infected cells and produce soluble factors that can limit HIV-1 spread. Despite years of searching, no single consistent T-cell functional or phenotypic marker has been associated with robust viral control. Rather, control is likely to be a combination of efficient recognition of peptide-loaded HLA molecules [9], broad target specificity [10], [11], rapid proliferation after exposure to cognate antigens [12], [13], efficient killing of infected cells [12], [13], [14], production of multiple soluble antiviral factors [12], [13] and the use of shared T-cell receptors or public clonotypes [15]. These T-cell functions are collectively measured in the viral inhibition assay (VIA), which quantifies reduction in HIV-1 replication in cultured autologous CD4^+^ T cells and does so in the context of immune response-evasive mechanisms [16]. Furthermore, VIA allows the use of diverse isolates including transmitted/founder viruses to assess the breadth of T-cell response coverage [17], [18], [19]. Indeed, HIV-1 inhibitory capacity *in vitro* predicted HIV-1 control *in vivo* by correlating with both viral load at set-point and the rate of CD4^+^ T-cell decline [20] as well as correlating with long-term elite virus control [21]. This makes VIA one of the most relevant and therefore critical assays for prioritizing T-cell vaccine strategies [17], [18], [19], [20], [21], [22], [23], [24], [25] prior to a phase IIb clinical efficacy study.

Analyses of T-cell specificity in chronic HIV-1 infection associated slow disease progression with Gag-specific T cells [10], [11], [26], [27] and their enhanced functional activity [28]. This is a consequence of the relative abundance and overall conservation of the Gag proteins, and their critical role in determining virus replicative capacity [29]. The importance of Gag was also demonstrated in the MRKAd5 vaccine Step study where broader Gag recognition was associated with a lower viral load in the event of HIV-1 infection [30], [31]. Several studies of natural chronic HIV-1 infection and limited vaccine efficacy in humans recommend the inclusion of Gag over other HIV-1 proteins into the HIV-1 T-cell vaccine formula. Despite this, vaccines vectored by human adenovirus alone or in a prime-boost with DNA expressing the full-length Gag have failed to induce responses that protect against HIV-1 acquisition [30], [32]: this may be a consequence of an overall suboptimal immunogen design, immunogen suboptimal delivery or both [33].

Recent work has suggested that a sub-protein definition of CD8^+^ T-cell specificities impacts on the level of HIV-1 viremia. In over 1000 treatment-naïve subjects infected with HIV-1 clades B or C, Mothe et al*.* identified responses to epitopes in Gag, Pol, Vif and Nef associated with high (bad epitopes) and low (beneficial epitopes) viral loads [34]. We and Rolland et al. [33], [35], [36] hypothesized that focusing vaccine-elicited T cells on the conserved regions of HIV-1 proteins would efficiently target both founder/transmitted and reactivated viruses and cause escape mutants to lose their replicative fitness [37], [38], [39]. Such conserved epitopes are typically subdominant in natural infection and an immunodominance hierarchy often undermines their protective potential and/or can completely preclude their detection [23], [40].

We constructed novel vaccine immunogen HIVconsv designed as a chimeric protein of alternating HIV-1 clade A, B, C and D consensus sequences of 14 highly conserved regions of the HIV-1 proteome [35]. In the first human trial HIV-CORE 002 testing HIVconsv vaccines in healthy HIV-negative individuals, we showed that conserved but subdominant epitopes, when taken out of the full-length protein context, induced strong CD8^+^ T-cell responses that inhibited replication of multiple HIV-1 variants in VIA [22]. The inhibition correlated with Gag and even more Pol-specific IFN-γ production [22]. Here, we further characterized the specificity and functionality of these inhibitory T cells in three vaccine recipients with the broadest viral control.

## 2 Materials and methods

### 2.1 Peptides and antigens

HIVconsv peptides (Ana-Spec, San Jose, USA) and their truncated versions (GenScriptHK, Hong Kong) were reconstituted to 10–40 mg/ml in DMSO and diluted to working stock solutions of 4 mg/ml in PBS. Recognized Gag- and Pol-derived peptides were assembled into personalized pools as described previously [22].

### 2.2 Expansion of Gag- and Pol-specific effectors

Effectors were expanded using either peptide pools or individual peptides as described in the legend of Supplementary Fig. S1A.

### 2.3 Viral inhibition assay (VIA)

Antiviral capacity of vaccine-elicited CD8^+^ T cells was assessed in VIA as described previously [21], [22]. Briefly, for each volunteer, autologous CD4^+^ T cells from a pre-vaccination time point were used as common targets for autologous CD8^+^ T-cell effectors from pre and post-vaccination time points. CD4^+^ and CD8^+^ T cells were first expanded for 7 days in R10 medium (RPMI 1460 supplemented with 10% FBS, 2 mM l-glutamine, 1 mM sodium pyruvate, 10 mM HEPES and penicillin-streptomycin antibiotics; Sigma Aldrich) supplemented with IL-2 at 50 IU/ml using CD3/8 and CD3/4 bi-specific antibodies, respectively, at 0.5 μg/ml (Donated by Prof. Wong, Harvard University). CD4^+^ T cells were washed, counted and infected with HIV-1 IIIB (clade B, CXCR4) and U455 (clade A, CXCR4). Viruses were added to the cell pellet at MOI 0.01. Cells were vortexed and placed at 37 °C, 5% CO_2_ for 3–4 h, washed twice in R10 and re-suspended at 1 × 10^6^/ml in R10/IL-2 at 100 IU/ml. Half a million cells were plated per well in a 48-well plate (Nunc; Sigma-Aldrich, UK). CD8^+^ T cells from each time point were washed, counted and re-suspended in R10 at 1 × 10^6^/ml and adding to the relevant CD4^+^ target wells (1:1 effector-to-target ratio if not specified otherwise). One well of infected CD4^+^ targets alone was also maintained for each virus, additional R10 was added to these wells to give a final R10/IL-2 concentration of 50 IU/ml. Cultures were fed on days 3, 6, 8 and 10 by the removal and replenishment of 0.5 ml R10/IL-2 at 50 IU/ml. On day 13, supernatants were collected and stored at −20 °C for p24 ELISA (PerkinElmer, Cambridge, UK). The log_10_ reduction in the p24 content in the supernatants of CD8^+^/CD4^+^ co-culture wells relative to infected CD4^+^ cells alone was calculated for each sample.

### 2.4 HIV-1 quantitative p24 ELISA

All frozen supernatant samples from the cultured VIAs were analyzed using an Alliance p24 ELISA kit (PerkinElmer, Cambridge, UK). Multiple dilutions of each sample were prepared to ensure that they fell within the standard curve. All samples were diluted in R10 medium and the assays were run according to the manufacturer's instructions. Plates were read at 492-nm with a 620-nm reference on BioTek ELx800 Absorbance Microplate reader with Gen5 software version 1.10.8 (BioTek Instruments Inc, Vermont, USA). Data analysis of the standard curve and p24 concentrations were determined in Microsoft Excel 2010 and for all cultured VIAs in GraphPad Prism version 5.04 (GraphPad Software, San Diego, CA).

### 2.5 Intracellular cytokine staining (ICS)

ICS assay was performed as described previously [22]. Methods are described in Supplementary Fig. S1B.

### 2.6 *In vitro* cell killing assay

Effectors from day 15 expansion were tested in an *in vitro* killing assay as described previously [41]. Methodological details are given in the legend of Supplementary Fig. S5.

### 2.7 IFN-γ ELISPOT assay

IFN-γ ELISPOT assay was performed as described previously [22]. Results were calculated by subtracting the average mock value from the peptide-stimulated wells.

## 3 Results

### 3.1 Vaccine-induced human Pol-specific CD8^+^ T cells inhibit HIV-1 replication *in vitro*

**Fig. 1:**
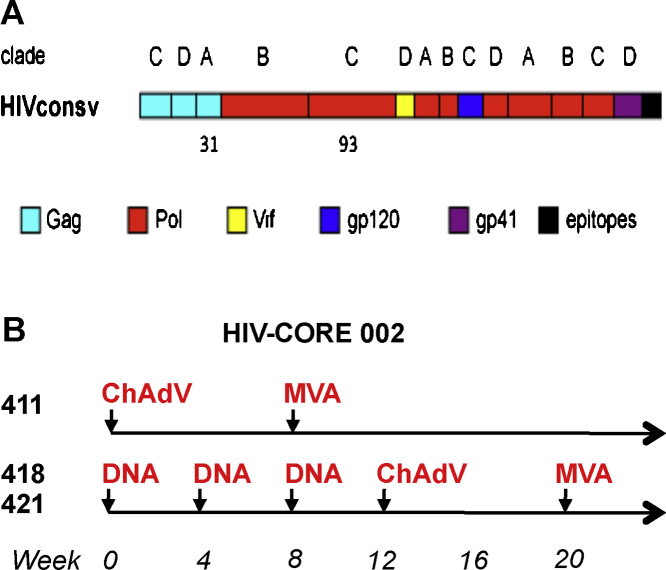
(A) A schematic representation of immunogen HIVconsv. Fourteen highly conserved clade consensus regions of HIV-1 proteins were assembled into a chimeric protein HIVconsv, whereby capital letters above bars indicate the clade used for the derivation of the consensus amino acid sequence [35]. The HIV-1 protein origins of conserved regions are color-coded. Approximate positions of peptides 31 and 93 are indicated. C-terminal epitopes (black) include immunodominant rhesus macaque and BALB/c CTL epitopes and mAb tag Pk facilitating pre-clinical monitoring. (B) The HIV-CORE 002 (COnserved REgions) [22] vaccinated a total of 23 volunteers in 4 arms (not shown). Volunteers 411, 418 and 421 concerned here received the indicated regimens of the following vaccines: DNA—4 mg of plasmid DNA pSG2.HIVconsv, ChAdV—5 × 10^10^ virus particles of non-replicating simian (chimpanzee) adenovirus 63-vectored vaccine ChAdV63.HIVconsv; and MVA—2 × 10^8^ non-replicating poxvirus-vectored vaccine MVA.HIVconsv. All vaccines were delivered by intramuscular needle injection into both arms.

**Fig. 2:**
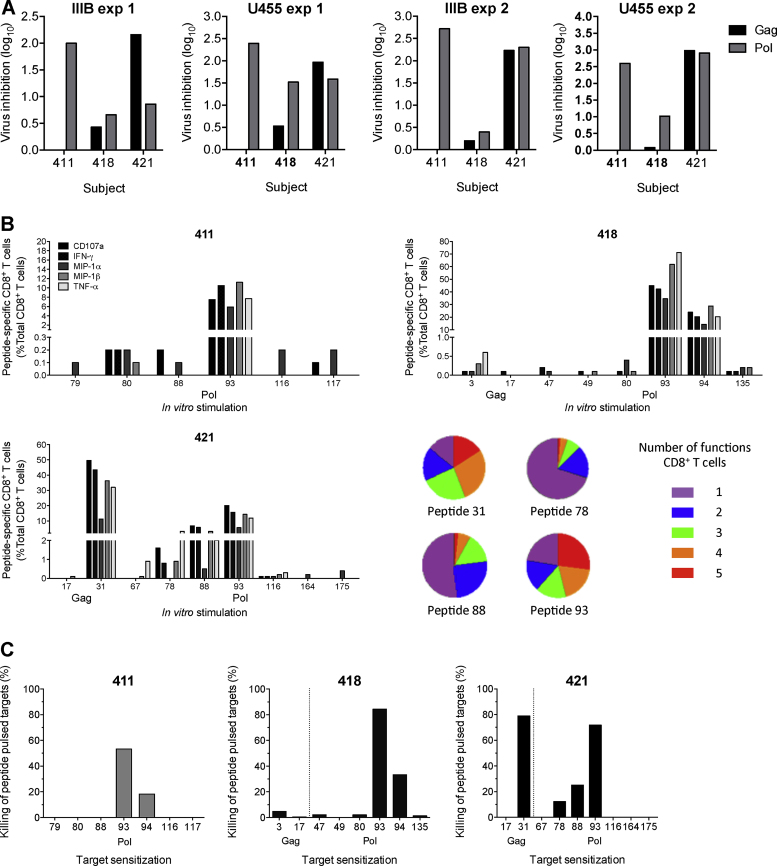
*In vitro* inhibition and functionality of HIV-1 by HIVconsv vaccine-elicited human effectors expanded using pools of cognate Gag and Pol-derived peptides. CD8^+^ T-cell effectors were expanded from frozen PBMCs of volunteers 411, 418 and 421 from 12, 1 and 2 weeks after the last vaccination with MVA.HIVconsv, respectively, using a pool of peptides mapped in IFN-γ ELISPOT assay (Table 1) [22]. The expansion is described in Supplementary Fig. S1A. (A) Gag (black) or Pol (grey) pool-expanded and sorted CD8^+^ T-cell effectors were incubated with autologous CD4^+^ T cells infected with HIV-1 IIIB and U455 and co-cultured for 13 days. HIV-1 in the supernatants was quantified using p24 ELISA. Background inhibition levels determined from placebo recipients are indicated by horizontal dotted lines. (B) The expanded cell functionality was determined in an ICS assay. Following a 6-h stimulation with individual peptides or medium alone, expanded CD8^+^ T cells were assessed for a variety of antiviral effector functions in stimulation to individual peptides, the HIV-1 protein origin of which is shown below the peptide number on the *x*-axis. Mock-stimulated background-subtracted cell frequencies for each function are shown. Dotted vertical lines separate Gag and Pol responses. Average number of functions over the three volunteers is summarized using pie charts. Sequential gating for cytokine and chemokine production is illustrated in Supplementary Fig. S1B. Oligofunctionality of cells was determined by Boolean gating in FlowJo and background correcting in Pestle is shown in Supplementary Fig. S2. Supplementary Fig. S3 shows pie charts of each volunteer/peptide. (C) The levels of peptide-specific killing by expanded human effectors. The sensitizing peptide numbers and their HIV-1 proteins of origin are indicated beneath each graph. Dotted vertical lines separate Gag and Pol responses. Peptide unpulsed lysis of targets (< 20%) was subtracted. The gating is illustrated in Supplementary Fig. S5.

Immunization of healthy HIV-negative volunteers with candidate HIVconsv vaccines (Fig. 1) induced effector CD8^+^ T cells capable of *in vitro* inhibition of a panel of HIV-1 viruses grown in autologous CD4^+^ T cells [22]. The initial experiments employed an antigen non-specific expansion of volunteers’ PBMCs using anti-CD3/CD4 bi-specific antibody [21], [22]. The frequencies of resulting expanded Pol-specific, IFN-γ-producing CD8^+^ T cells correlated significantly with the level of HIV-1-inhibition, but direct evidence for HIV-1 inhibition through recognition of Pol-derived epitopes was not formally obtained. Here, we aimed to determine the fine specificity of T cells responsible for HIV-1 inhibition. Volunteers’ PBMCs were first expanded using pools of either Gag- or Pol-derived 15-mer peptides overlapping by 11 amino acids (15/11) previously identified as stimulatory (Table 1) [22] and assessed in VIA for inhibition of HIV-1 IIIB (clade B, X4, nef-deleted laboratory isolate) and U455 (clade A, X4, laboratory isolate). These experiments were carried out using PBMCs from vaccine recipients 411, 418 and 421, who displayed the broadest HIV-1 inhibition, *i.e*. inhibited 8, 6 and 5 out of 8 tested viruses, respectively [22]. At the time points used for VIA here, volunteers 418, 411 and 421 had *ex vivo* broad responses of a total of respective 1605, 2260 and 1670 HIVconsv-specific cells per 10^6^ of PBMCs [22]. All three volunteers harbored Pol-specific effectors giving various levels of inhibition of the two tested viruses (Fig. 2A). However, only volunteer 421 showed a significant Gag-mediated inhibition of both viruses; volunteers 418 and 411, despite being broad controllers, had either detectable, but below background, or no inhibition at all through Gag epitopes, respectively. Thus, Pol-specific CD8^+^ T cells elicited in humans by strong vaccination regimens delivering conserved epitopes could inhibit HIV-1 replication *in vitro* at least as well as the Gag-specific effectors.

### 3.2 Expanded human CD8^+^ T cells recognizing Pol are oligospecific, oligofunctional and cytolytic

Specificities of the pool-expanded effector cultures tested in VIA were first deconvoluted in an ICS assay using single 15-mer peptides. Functionality of the CD8^+^ effectors was assessed for expression of IFN-γ, TNF-α, MIP-1α, MIP-1β and CD107a and multi-specific and strongly hierarchical Pol and Gag CD8^+^ T-cell responses were identified (Fig. 2B). For volunteers 411 and 418, these were dominated by Pol-derived peptide 93. Volunteer 421 had the broadest response of the three vaccine recipients with the strongest stimulation by Gag peptide 31 followed by Pol peptides 93, 88 and 78. In all three volunteers, responses to the other tested peptides were much weaker (Fig. 2B). For all strongly stimulatory peptides, the percentages of cells producing individual cytokines were similar, however, immunodominant CD8^+^ T cells recognizing peptides 31 and 93 were notably more oligofunctional than those responding to peptides 88 and 78 (Fig. 2B). Finally, effector cultures were tested for lysis of single peptide-pulsed autologous CD4^+^ T cells. For immunodominant peptides 31 and 93, the lysis at effector-to-target ratio 10:1 ranged from 53% to 84% (Fig. 2C). Overall, similar hierarchies in the lysis assay to those in the ICS assay were detected. Thus, in healthy human volunteers, the HIVconsv vaccines elicited relatively broad Gag- and Pol-specific CD8^+^ T cells capable of expansion upon stimulation with cognate antigens and lysis of peptide-pulsed CD4^+^ cells. Expanded responses were strongly hierarchical.

### 3.3 Virus inhibition by single-peptide expanded human effectors

**Fig. 3:**
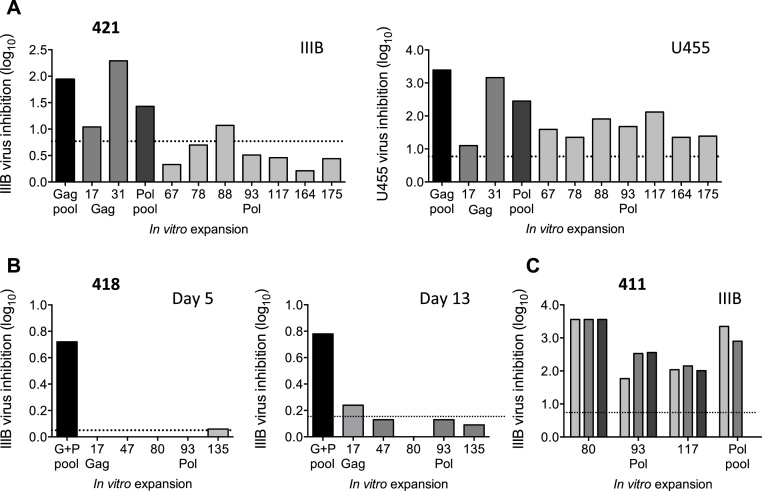
VIA efficiency of HIVconsv vaccine-elicited, single peptide-expanded human T-cells. Effectors were expanded from frozen PBMCs of HIVconsv vaccine recipients 411, 418 and 421 from 12, 1 and 2 weeks after the last vaccination with MVA.HIVconsv, respectively, using individual peptides mapped previously in the IFN-γ ELISPOT assay [22] and tested in VIA. (A) Effector cultures from volunteer 421 were tested for inhibition of HIV-1 IIIB (clade B) and U455 (clade A) viral isolates. (B) Volunteer 418 effectors used to assess the kinetics of inhibition by collecting VIA supernatants on days 5 and 13. (C) Expanded T-cells of volunteer 411 were tested in VIA at effector-to-target ratios of 1:1, the standard ratio (light grey), 2:1 (dark grey) and 3:1 (black). Peptide pool-stimulated samples served as positive controls (dark bars). Non-stimulatory peptide 22 or pool-stimulated pre-vaccination samples were used to determine the background inhibition (horizontal dotted line).

To demonstrate single-peptide specificities of vaccine-elicited human CD8^+^ T cells in VIA, effector cells were expanded by individual peptides. Thus, PBMCs from volunteer 421 were expanded with individual Gag and Pol peptides and co-cultured with autologous CD4^+^ cells infected with HIV-1 IIIB and U455. While only cultures expanded by peptide pools or the immunodominant individual peptides inhibited HIV-1 IIIB above the pre-vaccination levels, all conserved peptide cultures inhibited U455 (Fig. 3A). To decrease the background inhibition and, hypothesizing that viral inhibition in the assay wells occurs soon after combining the HIV-1-infected cells with effectors, inhibition by volunteer 418 was assessed early *i.e.* after 5 days, and at a standard time after 13 days of co-culture (Fig. 3B). While in the 5-day culture, inhibition was observed for the pool-expanded (multiple) effectors, single-peptide expanded cultures showed marginal inhibition above the background only after a 13-day culture for Gag peptide 17. Finally, for volunteer 411, all effectors expanded by single peptides 80, 93, 117 of Pol inhibited HIV-1 IIIB replication by 2 to 3.5 log_10_ in a 13-day assay and three effector-to-target ratios of 1:1 (the standard ratio), 2:1 and 3:1 displayed similar inhibitory levels (Fig. 3C).

**Fig. 4:**
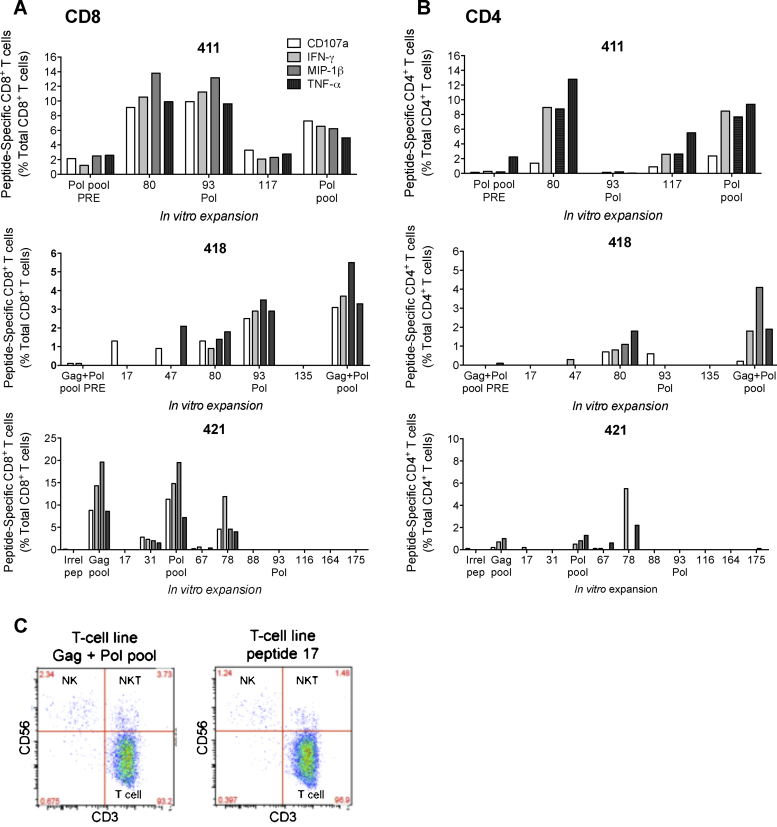
Functionality of vaccine-elicited single peptide-expanded human CD8^+^ and CD4^+^ T-cell effectors. Frozen PBMC samples from volunteers 411, 418 and 421 were expanded using single stimulatory peptides [22] for 10 days, rested in R10 and stimulated with the same peptide/pool for 6 h before staining for IFN-γ, TNF-α, MIP-1β and CD107a. No-peptide background subtracted percentages of CD8^+^ (A) and CD4^+^ (B) cells are shown. Peptide pool expansions and pre-vaccination samples were used as positive and negative controls. (C) All expanded effectors from volunteer 418 were analyzed for the NK (CD3^−^ CD56^+^), NKT (CD3^+^CD56^+^) and T-cell (CD3^+^CD56^−^) phenotypes to determine the frequencies of each population. The two FACS plots are representative of the phenotypic data for other peptide cultures generated from this volunteer.

The same *in vitro* expanded effectors were analyzed using polychromatic flow cytometry. Overall, the ICS dominant peptides corresponded to those observed in VIA and similar percentages of cells produced IFN-γ, TNF-α, MIP-1β and degranulation (Fig. 4A). In all three volunteers, this analysis also revealed some expansion of CD4^+^ cells (Fig. 4B). Cultures from volunteer 418 contained detectable levels of natural killer (NK) and natural killer T (NKT) cells expanded in both combined Gag + Pol pool and individual peptide cultures, but they were less than 4% of cultured cells (Fig. 4C). It is possible that these cells contributed to the viral inhibition.

### 3.4 Fine mapping of peptides recognized by human CD8^+^ T-cell effectors

**Fig. 5:**
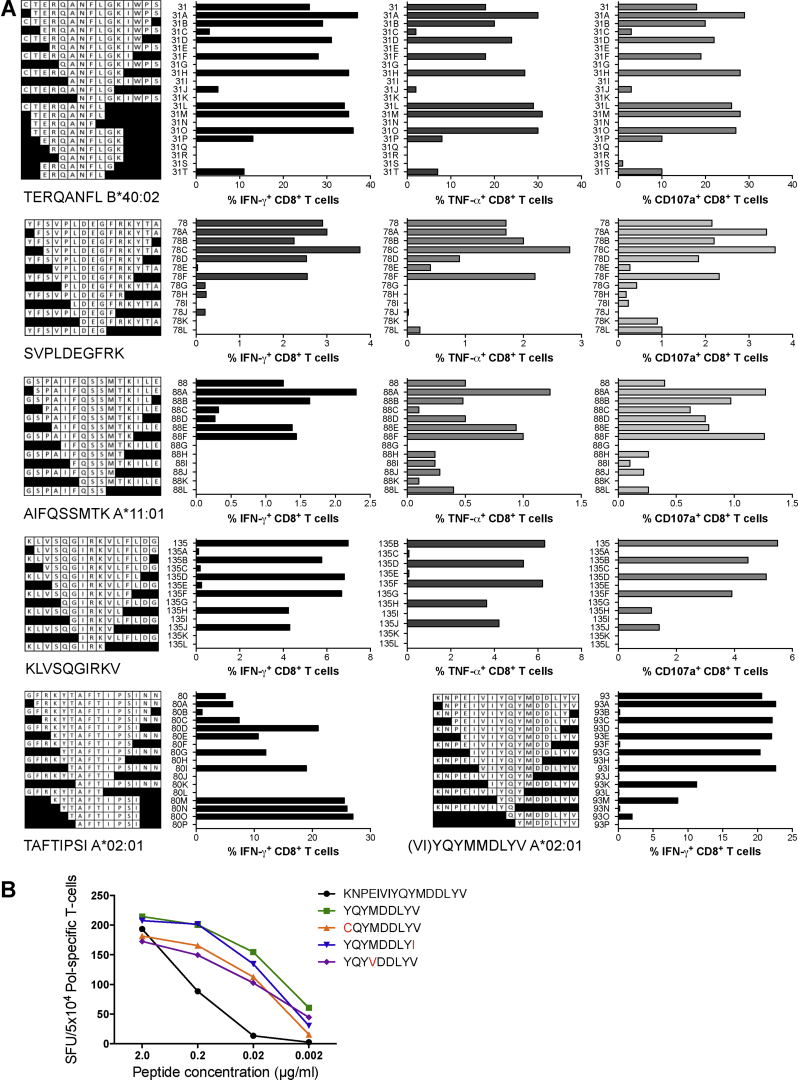
Minimum epitope mapping of HIVconsv vaccine-elicited, inhibitory CD8^+^ T-cell epitopes. (A) The minimum epitopes recognized by CD8^+^ T cells, which were elicited by the HIVconsv vaccines in human volunteers and mediated or contributed to the HIV-1 replication inhibition, were determined using the ICS assay (IFN-γ, TNF-α, and CD107a) and progressively truncated peptides. Known and volunteer-matching HLA class I-restricting molecules are given under the peptide maps (see also Table 2). (B) For the HLA-A*02:01-restricted YV9 epitope spanning the active site of HIV-1 reverse transcriptase, the recognition by volunteer 418 of titrated epitope variants was determined.

Populations of human effector cells expanded by a single 15-mer peptide were tested for recognition of progressively shorter peptides to identify the optimum stimulatory epitopes. For some epitopes, these were further confirmed by overlapping 7-, 8- and 9-mer peptides. Three functions of IFN-γ and TNF-α production and degranulation were monitored and closely agreed with each other in identifying the optimal stimulatory peptides (Fig. 5A). Thus, epitopes TERQANFL (TL8) and RQANFLGK (RK8) (peptide 31) of Gag and epitopes SVPLDEGFRK (SK10) (peptide 78), (KY)TAFTIPSI (KI10/TI8) (peptide 80), AIFQSSMTK (AK9) (peptide 88), (VI)YQYMMDLYV (VV11/YV9) (peptide 93) and KLVSQGIRKV (KV10) (peptide 135) of Pol were mapped, of which epitopes RK8, SK10 and KV10 are not present in the Los Alamos National Laboratory HIV Sequence Database (LANL-HSD) and were therefore considered novel. The predicted restricting HLA molecules and the detected CD4^+^ T-cell responses are summarized in Table 2. Relative frequencies of responding CD8^+^ and CD4^+^ T cells are shown in Supporting Fig. S4. The mapped epitope matches to the two viruses used in VIA are shown in Supplementary Table 1. Epitope (VI)YQYMMDLYV spans a highly conserved active site MDDL of the reverse transcriptase, which is present as YV9 in the database, however VY11 peptide provided a stronger stimulus. The YV9 epitope was dominant in all 3 HLA-A*02:01^+^ HIVconsv vaccine recipients and its 4 most common variants in the LANL-HSD were all similarly well recognized by CD8^+^ T cells elicited in volunteer 418 (Fig. 5B) further demonstrating the epitope's protective potential. Thus, when taken out of the full-length protein/virus context, conserved epitopes subdominant in natural HIV-1 infection can induce protective T-cell responses providing they are delivered by effective vaccine regimens.

## 4 Discussion

The work presented here further characterizes CD8^+^ T-cell effectors induced by conserved regions of the HIVconsv vaccines in HIV-1-negative adults [22]. Using samples from 3 vaccine recipients with the broadest inhibition of HIV-1 replication in trial HIV-CORE 002, we demonstrated that the inhibition *in vitro* happens through recognition of Pol-derived epitopes. Seven optimum CD8^+^ T-cell epitopes were mapped, of which 3 were not previously described in the LANL-HSD. Thus the HIVconsv vaccines elicited in humans CD8^+^ T cells capable of expansion upon encounter of cognate antigens, production of several cytokines, killing of peptide-pulsed targets and inhibition of HIV-1 replication *in vitro*. These are all highly desirable features of vaccine-elicited memory T cells, although perhaps not surprising in healthy immunocompetent vaccine recipients. While these observations do not question the key role for Gag-specific responses in protection against HIV-1 in chronic infection, they clearly show that, for an effective vaccine design, conserved regions of Pol, Gag and possibly other proteins should be harnessed.

Effector functions of vaccine-elicited human CD8^+^ T-cells were examined. Thus, T cells specific for peptides 93 (Pol) and 31 (Gag) effectively lysed peptide-pulsed targets and a large proportion also produced cytokines IFN-γ and TNF-α and β-chemokines MIP-1α and MIP-1β, which impede HIV-1 replication [42], [43]. Note that comprehensive analyses of released intercellular signaling molecules by the *ex vivo* volunteers’ PBMCs in trial HIV-CORE 002 were carried out previously [22]. For volunteers 411 and 418, T cells specific for peptide 93 dominated the ICS and killing assays suggesting that this was the main specificity contributing to viral control in these individuals. In volunteer 421, the dominant response was shared between T cells specific for peptides 31 (Gag) and 93 (Pol), but low-frequency T cells recognizing peptides 67, 78 and 88 (Pol) were also detected. HIVconsv epitope hierarchies were established in each vaccine recipient, but in contrast to natural infection, all epitopes were functionally conserved and likely contributed to HIV-1 control. The T-cell functional hierarchies of single peptide and pool-expanded cells did not match. The higher inhibition by pool-expanded cells suggests that a more effective control of HIV-1 may be dependent on a greater breadth of the CD8^+^ effector T-cell specificities concurring with the Step study post hoc analysis [30]. We also detected better inhibition of HIV-1 U455 than nef-deleted IIIB, despite minimal difference in the sequences of the mapped epitopes (Supplementary Table 1). This suggests that differences in inhibition between viruses are not only due to the degree of sequence conservation, but may reflect other as yet undefined virus properties [29], [44]. Perhaps by analogy to antibody inhibition, HIV-1 isolates can be classified to tier 1, 2 and 3 according to the ease of their inhibition by T cells [22].

During the HIV-1 lifecycle, there are fewer Pol transcripts produced with an estimated Gag-to-Pol ratio of 20:1 [45]. This limits the availability of Pol for presentation to T cells and likely causes a reduced T-cell priming by this protein. Importantly, the lower abundance of Pol did not prevent HIV-1-infected cells from being recognized through Pol subdominant epitopes [46], once the effectors were induced by strong vaccination.

Natural HIV-1 infection stimulates vigorous CD8^+^ T-cell responses, which do not clear HIV-1 infection nor prevent the development of AIDS [1], [4]. Therefore, aiming to induce ‘more of the same’ by vaccination will not be productive. The challenge is that the likely protective determinants are typically subdominant and overshadowed by dominant ‘decoy’ epitopes [33]. Thus, the goal of vaccination is to (re)direct effector CD8^+^ T cells towards conserved or ‘beneficial’ regions [33], [34], [35], [36], [47] and avoid the highly immunodominant variable decoy epitopes. Examples of the protective potential of subdominant epitopes are numerous both in animal models of viral infection and HIV-1 infection in humans [22], [23], [34], [47], [48], [49]. Clearance of the latent HIV-1 reservoir was also shown to require broad conserved cytotoxic T-lymphocyte responses due to the dominance of escape mutations established during chronic infection [37]. Thus, the conserved-region T-cell vaccine strategy complements antibody-inducing vaccines for prevention of HIV-1 acquisition while the antibody vaccines are suboptimal, and is likely key to HIV cure for elimination of cells with reactivated virus. In addition, conserved-region strategies may have broader applications for other variable pathogens where vaccine development faces similar challenges.

In summary, the work presented here together with emerging clinical data strongly supports a shift in T-cell HIV-1 vaccine design from full-length Gag protein-based vaccines to more refined, sub-protein immunogens directing vaccine-elicited T cells to conserved or ‘beneficial’ regions of Gag, Pol and, through extrapolation, to other such sensitive segments of the HIV-1 proteome. With the caveat of using samples from only 3 vaccine recipients, we demonstrated that subdominant Pol-specific CD8^+^ T-cell effectors can inhibit HIV-1 replication *in vitro* and that these inhibitory T-cells can be induced by vaccination. Inhibition is a qualitative feature with the VIA results being more representative of the situation *in vivo* where a variety of factors are integrated into the overall level of viral inhibition. The proportion of volunteers developing these effectors and frequency of these cells in their circulation are important, but separate issues, which can be addressed by the HIVconsv-like immunogen design [50] expression levels and more efficient ways of their delivery and presentation to the immune system influenced by the choice of vaccine vectors and their heterologous combinations/adjuvantation [33].

## Conflict of interest statement

The authors declare no financial or commercial conflict of interest.

## Figures and Tables

**Table 1:** Relevant stimulatory peptides mapped in cultured IFN-γ ELISPOT assay in HIV-CORE 002 (modified from Ref. [22]).

		Peptide	Volunteer no.		
No.	HXB2 aa position	Sequence	411	418	421
3	Gag 166-171	EVIPMFTALSEGATP	–	1200^a^	–
17	Gag 268-282	GLNKIVRMYSPVSIL	–	1583	517
31	Gag 426-440	CTERQANFLGKIWPS	–	–	683
47	Pol 154-168	LTQIGCTLNFPISPI	–	600	–
49	Pol 165-179	KNFPISPIETVPVKLK^b^	–	883	–
67	Pol 226/227-240	W-RKLVDFRELNKRTQ^c^	–	–	483
78	Pol 270-284	YFSVPLDEGFRKYTA	–	–	2367
80	Pol 277-293	GFRKYTAFTIPSINN	1917	667	–
88	Pol 311-325	GSPAIFQSSMTKILE	750	–	2017
90	Pol 319-333	SMTKILEPFRAQNPE	–	–	–
93	Pol 331-345	KNPEIVIYQYMDDLYV^b^	3967	4133	650
94	Pol 335-349	VIYQYMDDLYVGSDL	750	1300	–
116	Pol 563-577	ATWIPEWEFVNTPPL	617	–	–
117	Pol 567-581	PEWEFVNTPPLVKLW	1283	–	–
135	Pol 706-720	KLVSQGIRKVLFLDG	–	2050	–
164	Pol 891-906	VQMAVFIHNFKRKGGI	–	–	517
175	Pol 970-984	SDIKVVPRRKAKIIR	–	–	700

a Numbers indicate cultured IFN-γ ELISPOT assay frequencies as SFU/10^6^ PBMCs [22].

b Underlined K indicates lysine added to improve peptide solubility.

c ‘–’ dash in peptide sequence indicates junction between adjacent conserved regions.

**Table 2:** Mapped CD8^+^ T-cell epitopes for volunteers 411, 418 and 421.

Volunteer no	HLA	HIVconsv peptide	15-mer peptide	Mapped epitope	Short name	HLA LANL	IEDB binding predictions (HLA/score)
411	A*02:01/*02:01	Pol 80	GFRKYTAFTIPSINN	KYTAFTIPSI	KI10	A*02:01	–
B*08:01/*51:01			TAFTIPSI	TI8	B*51:01, A*02:01	B*51:01/0.6
Cw*03:03/*07:01	Pol 88	GSPAIFQSSMTKILE	Too low to map		CD4	–
Pol 93	KNPEIVIYQYMDDLYV	YQYMDDLYV	YV9	A*02:01	A*02:01/0.3

418	A*02:01/*24:02	Gag 3	EVIPMFTALSEGATP	Too low to map		CD4	–
B*07:02/*27:05	Gag 17	GLNKIVRMYSPVSIL	Too low to map		CD4	–
Cw*01:02/*07:02	Pol 93	KNPEIVIYQYMDDLYV	YQYMDDLYV	YV9	A*02:01	A*02:01/0.3
Pol 135	KLVSQGIRKVLFLDG	KLVSQGIRKV	KV10	None	A*02:01/6.25

421	A*02:01/*11:01	Gag 31	CTERQANFLGKIWPS	TERQANFL	TL8	A*40:02	A*40:02/1.1
B*35:03/*40:02			RQANFLGK	RK8	None	A*11:01/0.4
Cw*02:02/*12:03	Pol 78	YFSVPLDEGFRKYTA	SVPLDEGFRK	SK10	None	A*11:01/4.8
Pol 88	GSPAIFQSSMTKILE	AIFQSSMTK	AK9	A*11:01	A*11:01/0.1
Pol 93	KNPEIVIYQYMDDLYV	YQYMDDLYV	YV9	A*02:01	A*02:01/0.3
Pol 175	SDIKVVPRRKAKIIR	Too low to map		CD4	–

‘None’ in the HLA LANL column indicates previously undescribed minimum epitope; IEDB—immune epitope database, whereby the lower the score, the stronger the predicted binding.
