# Supplementary material for: Control of HIV-1 replication in vitro by vaccine-induced human CD8+ T cells through conserved subdominant Pol epitopes
Source: Vaccine. 2016 Feb 24;34(9):1215–24. doi: 10.1016/j.vaccine.2015.12.021 (PMC4769096; doi:10.1016/j.vaccine.2015.12.021)
Supplement: Supplementary file 1 [file mmc1.pdf]

Supplementary Table 1

Amino acid differences between the HIVconsv immunogen and HIV-1 isolates used in VIA

HIVconsv immunogen/peptides		Minimum epitope[a]	HIV-1 IIIB[b]	HIV-1 U455[b]
Gag 3	EVIPMFTALSEGATP	-	EVIPMF**S**ALSEGATP	EVIPMF**S**ALSEGATP
Gag 17	GLNKIVRMYSPVSIL	-	GLNKIVRMYSP**T**SIL	GLNKIVRMYSPVSIL
Gag 31	CTERQANFLGKIWPS	T**E**RQANF**L,** RQANFLGK	CTERQANFLGKIWPS	CTERQANFLGKIWPS
Pol 78	YFSVPLDEGFRKYTA	SVPLDEGFRK	YFSVPLDE**D**FRKYTA	YFSVPLDE**S**FRKYTA
Pol 80	GFRKYTAFTIPSINN	T**A**FTIPSI	**D**FRKYTAFTIPSINN	**S**FRKYTAFTIPSINN
Pol 88	GSPAIFQSSMTKILE	AIFQSSMT**K**	GSPAIFQSSMTKILE	GSP**S**IFQSSMTKILE
Pol 93	KNPEIVIYQYMDDLYV	Y**Q**YMDDLY**V**	QNPDIVIYQYMDDLYV	**QH**P**D**IVIYQYMDDLYV
Pol 135	KLVSQGIRKVLFLDG	KLVSQGIRKV	KLVS**A**GIRKVLFLDG	KLVS**S**GIRKVLFLDG
Pol 175	SDIKVVPRRKAKIIR	-	SDIKVVPRRKAKIIR	SDIKVVPRRKAKIIR

a – Bold underlined aa indicate anchor residues for restricting HLA molecules

b – Bold underlined aa indicate differences between the vaccine and the HIV-1 isolates used in VIA.

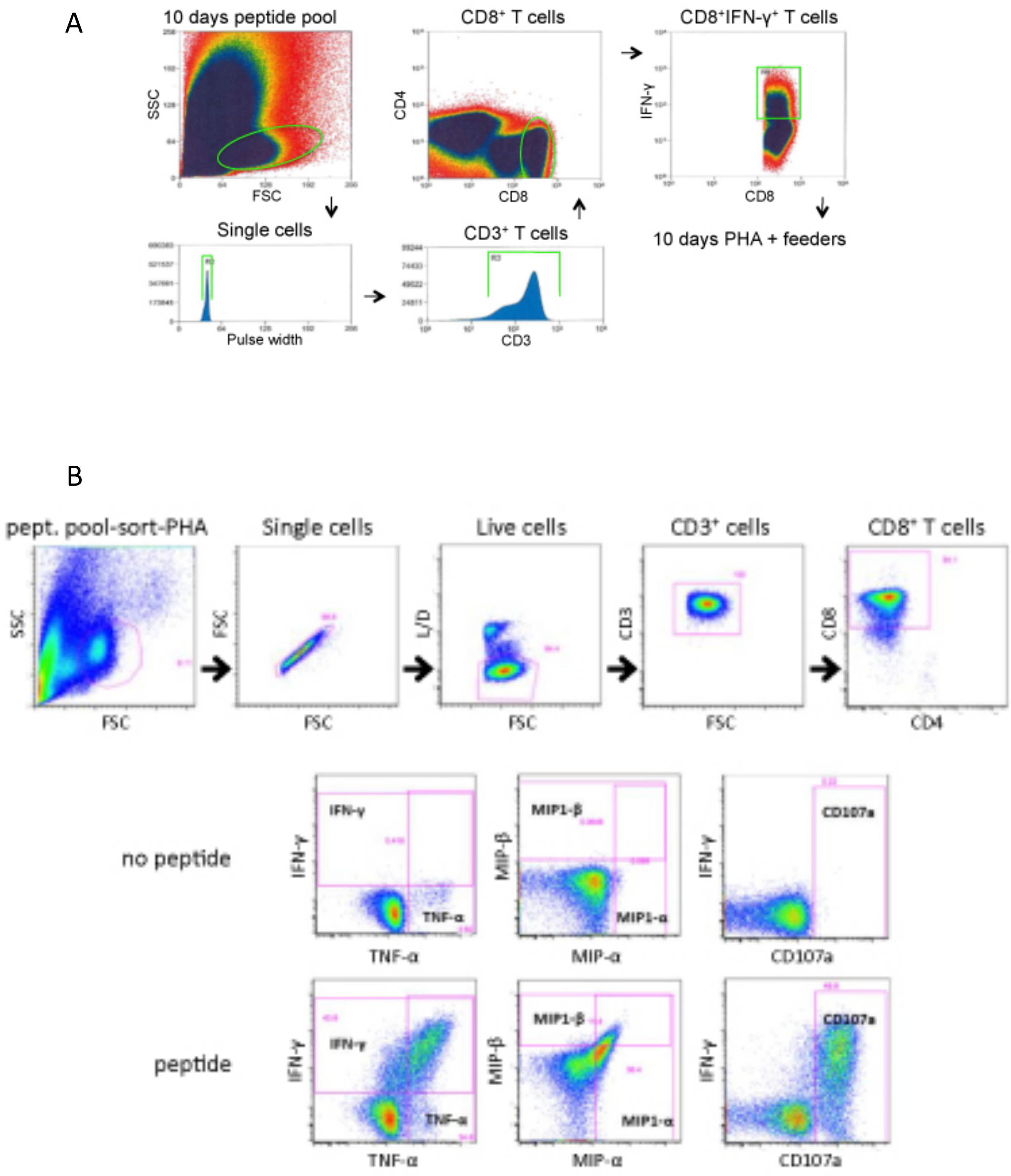
A
10 days peptide pool
SSC
FSC
Single cells
Pulse width
CD3⁺ T cells
CD3
CD8⁺ T cells
CD4
CD8
CD8⁺IFN-γ⁺ T cells
IFN-γ
CD8
10 days PHA + feeders
B
pept. pool-sort-PHA
SSC
FSC
Single cells
FSC
FSC
Live cells
L/D
FSC
CD3⁺ cells
CD3
FSC
CD8⁺ T cells
CD8
CD4
no peptide
peptide
IFN-γ
TNF-α
MIP1-β
MIP1-α
MIP-β
MIP-α
CD107a

Suppl Fig. S1

Supplementary Fig. S1. (A) Expanded human effectors were derived from frozen PBMC samples. The PBMC were thawed and equal volume of R10 plus 50 U/ml benzonase nuclease (Novagen, Houston, USA)) warmed to 37 °C was added dropwise. The cells re-suspended at a concentration of 1 to 3 x10^6 cells/ml in R10 with IL-7 at 25 ng/ml. To each well, 900 µl of cells along with 100 µl of 15 µg/ml peptides were plated, the cells and antigen were gently swirled to mix, PBS was added to empty wells to maintain humidity and the plates were incubated at 37 °C, 5% CO_2 for 10 days. On day 3 cells, were fed with IL-2 at a final concentration of 100 IU/ml. On day 7, R10 media and IL-2 were added to the plate to give 100 IU/ml of IL-2 in 2 ml. On day 10, the cells were washed 3x with R10, re-suspended in 1 ml of R10 and placed with loose lids at 37 °C, 5% CO_2 for 48 hours to rest. Cells were stimulated for 3 hours with the peptide pool (10 µg/ml), and CD8+ IFN-γ producing cells identified using the IFN-γ capture assay, (130-054-202; Miltenyi Biotec, Bisley, UK) together with cell surface staining with anti-CD8 APC, anti-CD3 APC-Cy7 and anti-CD4 PerCP to facilitate HIV-1-specific $CD8^+$ T-cell isolation. IFN-γ producing $CD3^+CD4^-CD8^+$ cells were sorted using a MoFlo cell sorter (DakoCytomation, Glostrup, Denmark). Feeder cells from 3 healthy donors were prepared on the day of cell sorting, re-suspended in 10 ml R10 and placed in 25-cm^2 tissue culture flasks for irradiation at 50 gray per flask. Once irradiated, the cells were mixed in a 50 ml falcon tube and washed with 50 ml R10 at 1,500 rpm for 10 minutes, resuspended and diluted to 2x10^6/ml in R10 and PHA (40 µg/ml), and incubated at 37 °C, 5% CO_2 until sorting. Once Gag- and Pol-specific cells were sorted into 100 µl of R10, 1 ml of feeder cells was added and the total volume was plated into a single well of a 24-well flat-bottomed tissue culture plate and maintained at 37 °C, 5% CO_2. Expanded cells were fed with R10/IL-2 at 200 IU/ml as required, or IL-2 alone if growth was slow. On day 13, expanded cells were re-stimulated using irradiated PBMC prepared as described above. Cell lines were transferred to 15-ml falcon tubes, washed in 10 ml R10 and counted. Cell lines were then pelleted by centrifugation at 1,500 rpm 10 minutes, feeders were added directly to give a feeder-to- ratio of 2:1 and the cultures were then plated into 12-well plates and fed with R10/IL-2 at 200 IU/ml as required. On day 14, expanded cells were ready for use and a sample was stained for cell purity and antigen specificity. Day-14 expanded cells were tested by cultured VIA.

(B) An example of sequential gating for cytokine and chemokine production shown in Fig. 2B and C. Gag- and Pol-specific expanded effectors were resuspended in R10 medium at 10^6 cells/ml. The cells were labelled with anti-CD107a-PE-Cy7 (BD Biosciences, Hatfield, UK), stimulated with 1.5 µg/ml of each individual peptide, 1 µg/ml SEB (positive control) or R10 (negative control) and 1 µg/ml of anti-CD28 and anti-CD49d. Then, 10 µg/ml of GolgiPlug (BD Biosciences, San Jose, USA) and 0.7 µl/ml of GolgiStop (BD Biosciences, San Jose, USA) were also added and plates were incubated for 6 hours at 37 °C, 5% CO_2. The next day, cells were stained with the surface antibodies anti-CD8 APC-efluor780 (47-0087-42; eBioscience, San Jose, USA), anti-CD4 BV570 (300533; Biolegend, London, UK) and Aqua Live/Dead (Invitrogen, Grand Island, USA) for 30 minutes at 4 °C. Cells were then washed and permeabilized with cytofix/cytoperm solution (BD Biosciences, San Jose, USA) and intracellular staining was performed using anti-MIP-1α PerCP-eFluor 710 (46-7539; eBioscience, Hatfield, UK), anti-MIP-1β-Alexa Fluor 700 (561278; BD Biosciences, San Jose, USA), anti-TNF-α APC (562084; BD Bioscience, San Jose, USA), anti-IFN-γ V450 (560371; BD Biosciences, San Jose, USA) and anti-CD3 ECD (A07748; Beckman Coulter, Dustable, UK) for 30 minutes at 4 °C. Cells were then washed twice in FACS buffer and fixed in 1% paraformaldehyde before being acquired on the BD LSRII flow cytometer (BD Biosciences, San Jose, USA). Data analysis was performed using FlowJo software (Tree Star Inc., Ashland, USA) version 9.5.

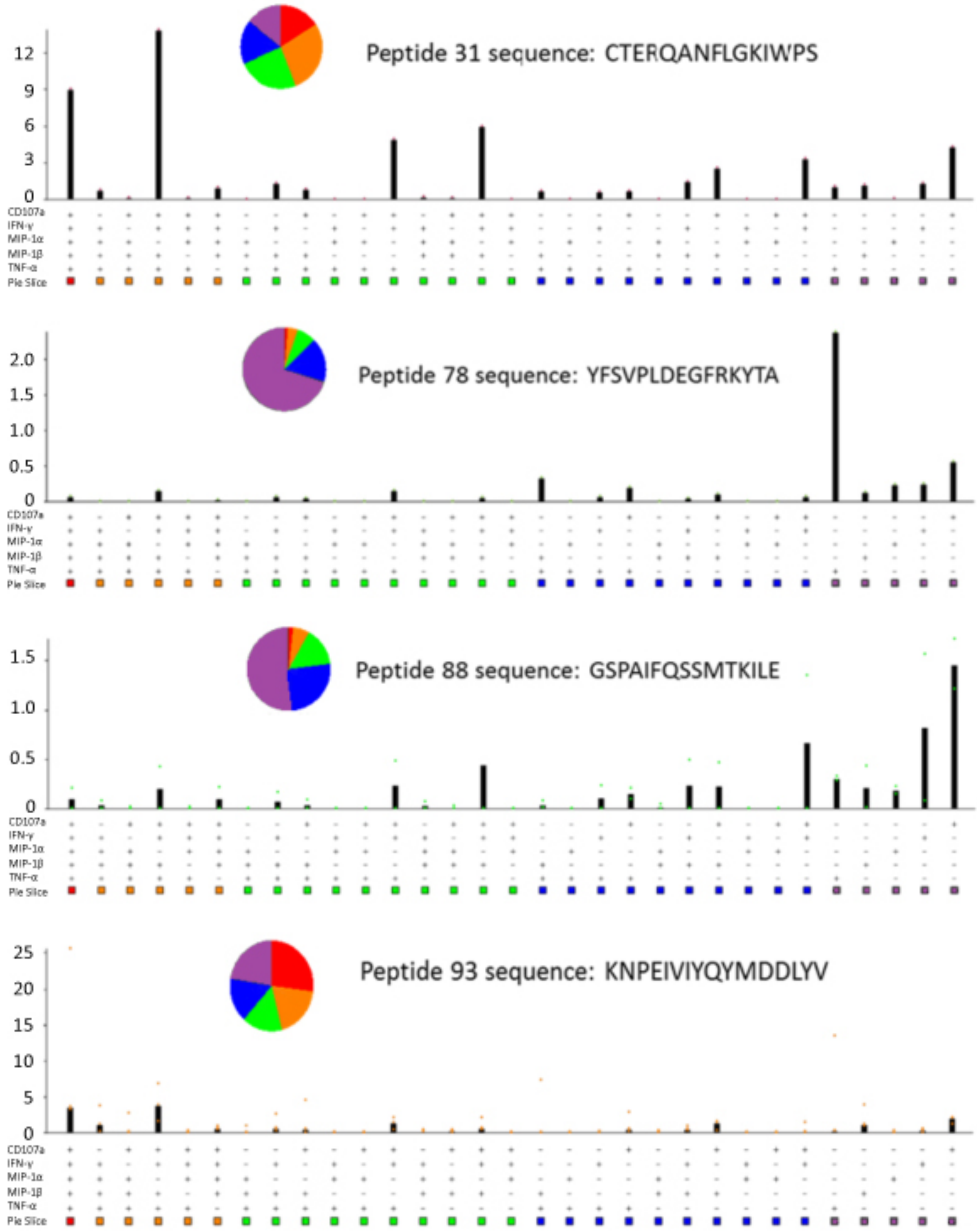

Supplemetary Fig. S2. SPICE graphs for average oligofunctionality of HIVconsv vaccine-induced, Gag and Pol pool-expanded human effectors specific for individual peptides. Rested STCL cultures were stimulated with individual peptides in an ICS assay. Peptide sequences are shown above each graph. Number of functions: 1 – purple, 2 – blue, 3 – green, 4 - orange and 5 – red.

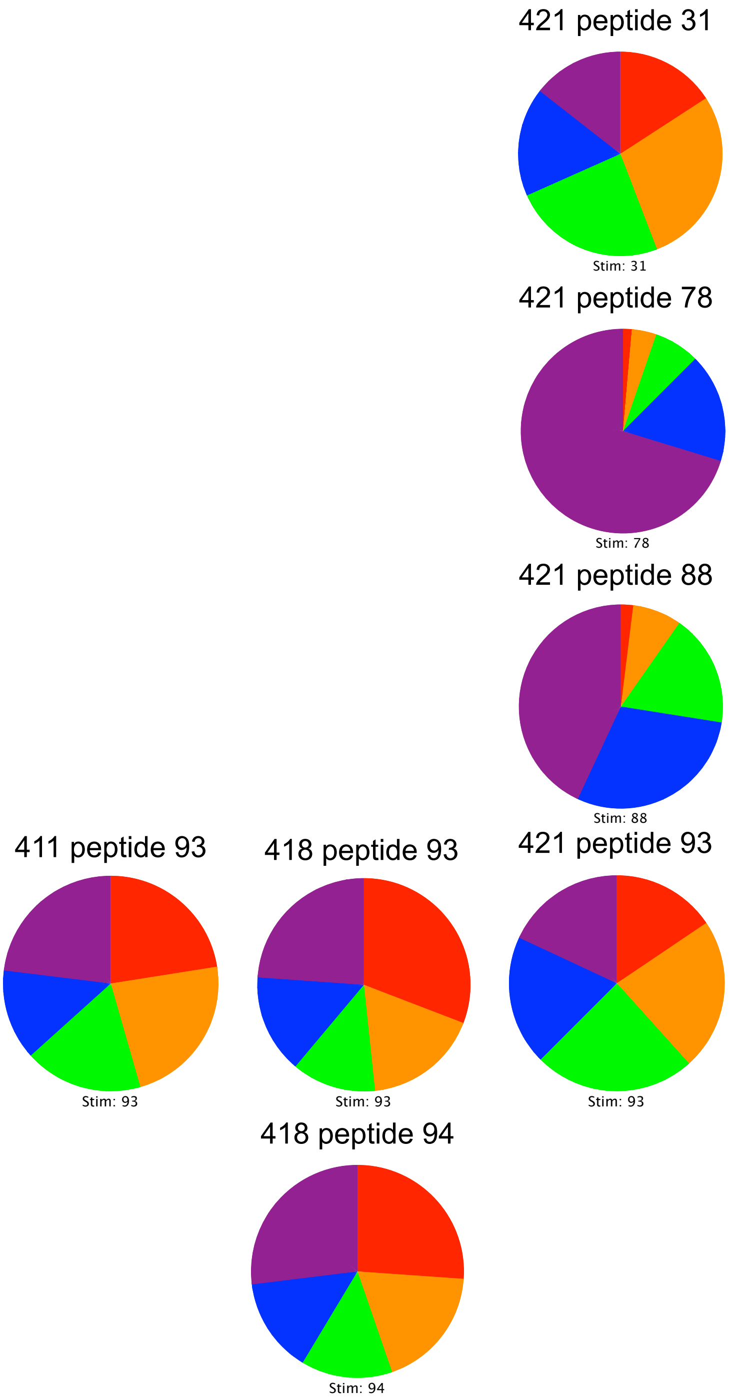

Supplementary Fig. S3. Pie charts depicting oligofunctionality of Gag and Pol pool-expanded human effectors induced in volunteers 411, 418 and 421 specific for individual peptides. Rested short-term cell cultures were assessed in an ICS assay for their specificity and functionality using a single peptide restimulation. Volunteer and peptide numbers are shown above charts. Number of functions: 1 – purple, 2 – blue, 3 – green, 4 -orange and 5 – red.

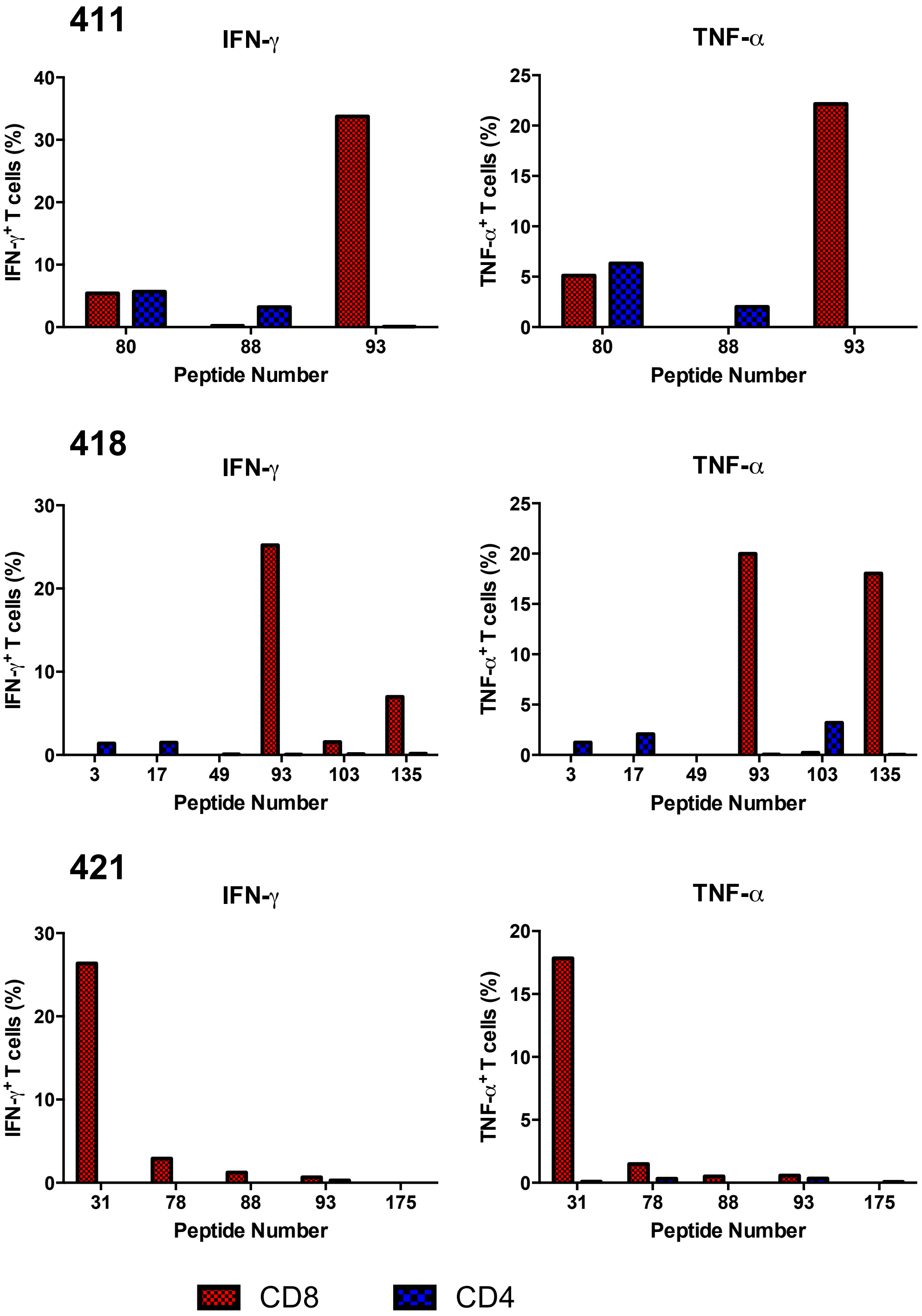

Supplementary Fig. S4. Relative frequencies of single peptide-expanded CD8+ and CD4+ T-cell responses. HIVconsv vaccine-elicited human CD8+ T cell effectors were expanded using individual 'parental' 15-mer peptides. Cells were rested and then stimulated with progressively truncated peptides for 6 hours as shown in Fig. 6a. The frequencies of strongest short-peptide responses are shown.

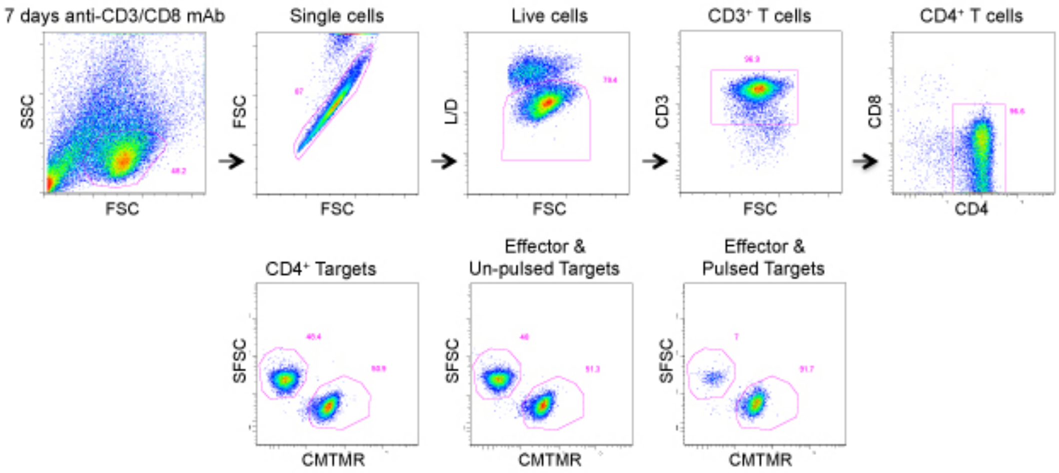

Supplementary Fig. S5. Determining the level of killing in Fig. 3. Gag- and Pol-specific expanded human effectors from day 15 were tested in an *in vitro* cell killing assay. Briefly, $CD4^+$ T cells expanded by anti-CD3/CD8 bi-specific antibody were separated into 1x 50 ml falcon tube for pulsed cells, and 2x 15-ml falcon tubes for un-pulsed and unstained cells. Cells for peptide-pulsing were re-suspended at $2x10^6$/ml in PBS and were stained with 8 nM CFSE (65-0850-84; eBiosciences, Hatfield, UK) by adding an equal volume of CFSE stain to cell suspension for 10 minutes in the dark at 18-24 °C. Un-pulsed cells were stained with cell tracker orange, CMTMR (C2927, Invitrogen, Grand Island, USA) reconstituted to a 10 mM solution and diluted by adding 8 µl into 10 ml R10 to give a working stock. Samples were placed, with loose lids at 37 °C, 5% CO_2 for 12 minutes, after which they were centrifuged to remove the stain and re-suspended in R10 at $2x10^6$/ml before being place back at 37 °C, 5% CO_2 for a further 15 minutes. For all cells, the reaction was quenched by 5x volume of FCS added to all tubes for 5 minutes on ice. Cells were then washed 3x in R10, re-suspended in R10 at $2x10^6$/ml and counted by trypan blue exclusion dye. CFSE-stained cells were then divided into relevant number of tubes for pulsing with individual peptides and un-pulsed CFSE-labeled cells were included as a control. Cells were centrifuged and supernatant removed, peptides were added at 2x concentration (3 µg/ml), directly to the re-suspended cell pellet and all samples were placed with lids loose at 37 °C, 5% CO_2 for 2 hours, washed 3x and re-suspended at $1x10^6$/ml and added to assay wells to give 50,000 pulsed and 50,000 un-pulsed per well. Effector Gag- and Pol-specific $CD8^+$ T-cell lines were washed, diluted to 10^7 cells/ml and added into assay wells to give a 10:1 effector to target ratio. All wells were made to 200 µl volume and empty wells were filled with PBS. The plate was cultured overnight at 37 °C, 5% CO_2. The next day, cells were washed, re-suspended in 200 µl FACS buffer, stained with Live/Dead Violet (L34955; Invitrogen, Grand Island, USA) and anti-CD8 APC for 20 minutes at 18-24 °C in the dark, washed in 200 µl FACS buffer and re-suspended in 200 µl 1% paraformaldehyde and acquired on the CyAn ADP flow cytometer (Beckman Coulter, Dustable, UK). Data analysis was performed using FlowJo software version 9.5 (Tree Star Inc. Ashland, OR, USA).
